# Palatable Castor Oil

**Published:** 1880-02

**Authors:** 


Palatable Castor Oil.—Rub two drops of oil of cinnamon with an ounce of glycerine, and add an ounce of castor oil. Children will take this well and ask for more.

